# Is age of self-harm onset associated with increased frequency of non-suicidal self-injury and suicide attempts in adolescent outpatients?

**DOI:** 10.1186/s12888-022-03712-w

**Published:** 2022-01-26

**Authors:** Anne Brager-Larsen, Pål Zeiner, Ole Klungsøyr, Lars Mehlum

**Affiliations:** 1 Child and Adolescent Mental Health Research Unit, Department of Research and Innovation, Division of Mental Health and Addiction, Oslo University Hospital, Sognsvannsveien 12, Bygg 12, N-0372 Oslo, Norway; 2 Section for treatment research, Department of Research and Innovation, Division of Mental Health and Addiction, Oslo University Hospital, Oslo, Norway; 3 National Centre for Suicide Research and Prevention, Institute of Clinical Medicine, University of Oslo, Oslo, Norway

**Keywords:** Self-harm, Suicide attempt, Non-suicidal self-injury (NSSI), Adolescents, Age of onset, Borderline symptoms

## Abstract

**Background:**

Self-harm in adolescents is an increasing public health concern and an important risk factor for suicide. We aimed to examine how age of self-harm onset in adolescents was associated with frequency of subsequent suicidal and non-suicidal self-harm (NSSI) episodes, and how age of onset and duration of self-harm may influence the frequency of self-harm.

**Methods:**

Data from 103 adolescents with recurrent self-harm recruited from child and adolescent psychiatric outpatient clinics were collected through clinical interviews and self-reports, and analysed with negative binomial and hurdle models.

**Results:**

A lower age of self-harm onset and a longer duration of self-harm were both significantly associated with increased frequency of subsequent episodes of NSSI and risk of a first suicide attempt. There was an increase in repeated suicide attempts when the age of onset of self-harm decreased and the duration increased, and dramatically more for long duration of NSSI before first suicide attempt.

**Conclusion:**

Initiating self-harm behaviour at the youngest age had the highest risk of increased frequency of NSSI and suicide attempts. Longer duration of self-harm behaviour increased this risk. This underlines the importance of early identification of self-harm behaviour in adolescents, and the use of effective interventions.

## Background

Suicide attempts and non-suicidal self-injury (NSSI) (both subsumed “self-harm”) in adolescents are public health concerns [1, 2]. Both suicide attempts and NSSI in adolescence are risk factors for future suicidal behaviour [3–5]. Self-harm behaviour increases up to age 12 and peaks between ages 14–16, with decreasing rates after 18 years of age [6, 7]. In a study of self-harm prevalence among Norwegian adolescents they found a 4-fold increase in prevalence over a 15-year period [8]. The age with highest probability of NSSI onset is between 14 and 15 years, suggesting that adolescence is the most critical period for such behaviour [7]. Determining whether an early age of self-harm onset predicts increasing self-harm behaviour, merits considerable interest. Understanding how different ages of self-harm onset may be associated with a later course of NSSI and suicide attempts, could help us target risk groups in clinical settings. Ammerman et al. [9], found that onset before the age of 12 was associated with an increased lifetime frequency of NSSI episodes and more severe self-harm behaviour. Whether the age of onset was totally or partially a function of duration, however, was not investigated. Muehlenkamp et al. [10], similarly found that self-harm onset before the age of 12 was associated with a significantly higher lifetime frequency of NSSI episodes compared to participants with an onset at older ages, even when controlled for duration. These studies were both based on general population samples and are not readily generalizable to adolescent clinical populations.

Studies focusing on duration of self-harm behaviour have found that longer duration is associated with increased frequency and severity of self-harm behaviour [11, 12]. Although these studies did not address how the different ages of onset in children and adolescents may affect the frequency of self-harm behaviour, the results illustrate that the duration of self-harm is an important factor to consider when assessing age of self-harm onset in relation to frequency of NSSI and suicide attempts.

Adolescence is an important developmental time-period and the prevalence of many psychiatric illnesses increases sharply from childhood to adolescence [13], as is also the case with self-harm behaviour, making adolescence a very important time-period for prevention. However, we still lack knowledge on how initiating self-harm behaviour at earlier or later ages may give rise to different self-harm trajectories. Thus, there is a need for studies to investigate if the age of self-harm onset and duration of self-harm, affect the following clinical course of self-harming behaviour.

To investigate if age of onset and duration, uniquely and in interaction, are associated with frequency of NSSI and suicide attempts in an adolescent psychiatric population, it is essential to also consider other relevant factors. We know that factors such as history of sexual or physical abuse, [14], relationship with parents, [15, 16], and borderline symptoms, [17, 18], are strongly associated with both NSSI and suicide attempts, and age of onset. We also know that these factors are commonly observed in clinical adolescent populations [19, 20]. What we do not know is whether these factors affect how age of self-harm onset and duration of self-harm are associated with frequency of NSSI and risk of suicide attempts.

Given the current state of knowledge, we wanted to study if age of self-harm onset is associated with increased lifetime frequency of NSSI episodes, uniquely and in interaction with duration of self-harm, and adjusted for covariates. We also wanted to study if age of self-harm onset, uniquely and in interaction with duration of self-harm, is associated with an increased risk of having made a suicide attempt and of having repeated suicide attempts, adjusted for covariates. We hypothesized that lowered age of self-harm onset was associated with increased lifetime frequency of NSSI episodes and risk of first and repeated suicide attempts, uniquely and in interaction with duration of self-harm, adjusted for covariates.

## Method

### Participants and procedures

Participants were 103 adolescents (age 12–18) recruited from a child and adolescent psychiatric outpatient clinic at Oslo University Hospital, Norway, serving a catchment area in the city of Oslo, with mixed social classes and socioeconomics. Adolescents were screened for self-harm behaviour. Self-harm was defined as “intentional poisoning or self-injury, regardless of intention to die” [21]. Suicide attempt was defined as “a potentially self-injurious act committed with at least some wish to die, as a result of the act” [22], while non-suicidal self-injury (NSSI) was defined as “the deliberate, self-inflicted destruction of body tissue without suicidal intent, and for purposes not socially sanctioned” [23]. Inclusion criteria were recurrent self-harm behaviour (two or more episodes) with the last episode having occurred within the past 6 months. Exclusion criteria were intellectual disability or insufficient Norwegian language skills to understand or answer the interviews or questionnaires. All patients signed a consent declaration to participate in the study. The declaration was approved by Regional Committee for Medical Research, South-East Norway. The interviewers were experienced clinicians, having received training and supervision in the use of the study instruments.

### Measures

The *Suicide Attempt Self-Injury Interview (SASII)* [24], was used to collect data on age of self-harm onset, frequency of non-suicidal self-injury episodes (NSSI), first and lifetime suicide attempts and methods in use. The SASII-timeline was used to guide the exploration of when, how and how often the individual harmed themselves. The SASII is a highly robust and comprehensive instrument with good psychometric properties [24, 25]. The *Schedule for Affective Disorders-Present and Lifetime version, 2013 (K-SADS-PL)* [26], was used to obtain socio-demographic data and DSM-5 Axis I diagnoses, and is a semi-structured highly robust and comprehensive interview, with excellent interrater reliability [27]. *Childhood interview for Borderline Personality Disorder (CI-BPD),* was used to assess BPD criteria based on *DSM-5*, with good psychometric properties [28]. The self-report *Borderline Symptom List (BSL-23)* was used to assess borderline-specific symptoms, and has shown good psychometric properties [29]. Finally, relationship to parents (attachment) was measured through a shortened version of the self-report *Inventory of Parental and Peer Attachment (IPPA)* [30] while *Previous experience of sexual and physical abuse* was assessed using the CARE instrument [31].

### Statistics

Means and standard deviations or median and interquartile ranges (IQR) were computed for normally and non-normally distributed variables respectively (Table 2). As is commonly observed in clinical studies of self-harm, number of NSSI episodes and suicide attempts showed over dispersion leading to more zeros and heavier tails than in a Poisson-model [32]. Therefore, for NSSI as outcome, a negative binomial regression model was fitted and the results are presented in both table and plot of predicted mean frequency of NSSI episodes. For first and repeated suicide attempts as outcome, a two-component “hurdle” model was fitted [32, 33]. One component models zero versus positive counts, where a logistic regression model (logistic link) was employed. The other component is a truncated count component for positive counts, where a negative binomial regression (log link) model was employed. Since all participants had self-harm behaviour, the hurdle model was appropriate for suicide attempts only. Regression results from each component are presented in tables. Predicted mean frequency of suicide attempts was calculated based on the results from the total model (both first and repeated suicide attempts), and plotted. Significant covariates in either component is significant in the total hurdle model. Maximum likelihood estimation was achieved with the function glm.nb() from the R-package MASS and the function hurdle() in the R-package pscl [34].

**Fig. 1:**
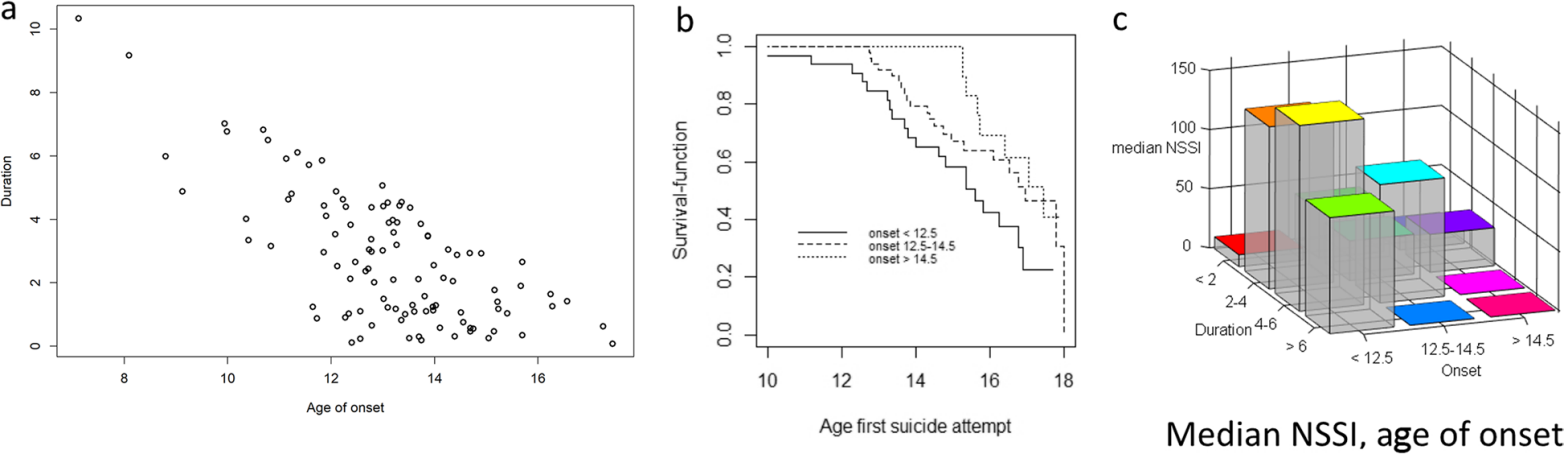
**a** Variation in age of self-harm onset, for each level of duration of self-harm. **b** Kaplan-Meyer plot of age at first suicide attempt for three strata of age of self-harm onset, **c** 3d bar plot with group median NSSI lifetime episodes for three strata age of self-harm onset and four strata duration of self-harm, in adolescents with repetitive self-harm (N = 103)

The variation in age at time of interview made it possible to model both the effect of age of onset and duration of self-harm (Fig. 1a). For each value of duration, there was a spread in the age of onset, and vice versa. Without this variation (fixed age at interview), they would be linearly dependent and the effects would not be separable. For illustrative purposes, we assessed group differences in time to first suicide attempt and median NSSI frequency (Fig. 1b and c).

Age of onset was defined as the age (years) at the time of first self-harm episode. Total duration of self-harm was defined as the time-span between the age of onset and the time of interview. When analysing risk of suicide attempts, duration was defined as “duration of NSSI before first suicide attempt” (if any). Age at time of interview was considered to reflect number of years under risk, and was entered as an offset-term in all regression models.

### Adjusting for covariates

To assess direct/indirect effects (as well as effect-modification) of age of onset through duration would be interesting, but rely on complete set of measured confounders [35, 36]. With duration as a mediator between age of onset and suicide attempts, confounders for duration and suicide attempt should be adjusted for [36]. In the present study, no attempt was made to do causal inference, but rather to indicate mechanisms, and the associations found must be interpreted with this in mind. Selected covariates that may influence the association between the age of onset, separately and in interaction with duration, as well as the outcome variables, were sexual and physical abuse, borderline symptoms (BSL-23) and relationship with parents. These covariates are thought to precede age of self-harm onset and duration of self-harm. All demographic and clinical characteristics were analysed using the IBM SPSS statistics version 25, on descriptives and frequencies. All regression analyses and figures were conducted with the R statistical software [37], and the significance level was set to .05.

## Results

### Sample characteristics

A total of 103 adolescents with a mean age of 15.9 years (SD = 1.47) were enrolled in the study between January 2017 and June 2019. Mean number of DSM-5 diagnoses was 3.2 (range = 0–8) with mood disorders (84.5%) and anxiety disorders (73.8%) as the most frequent. Nearly one third (28.2%) of the adolescents fulfilled diagnostic criteria for Borderline personality disorder. Sociodemographic and clinical characteristics are shown in Table 1.

**Table 1:** Sociodemographic and clinical characteristics of adolescents with repetitive self-harm (*N* = 103)

	Number	Percent
Gender (females)	89	86.4
Age in years, (mean, SD) (15.9, 1.47)		
Born in Norway	87	84.5
One or both parents born in foreign countries^c^	43	41.7
Living with - both parents	39	37.9
- alternating between parents	15	14.6
- single parent	34	33.0
**Current DSM-5 diagnosis**^a^		
Mood Disorder, any	87	84.5
Psychotic Disorder, any	13	12.6
Anxiety Disorder, any	76	73.8
Eating Disorder, any	18	17.5
Substance-Use Disorder, any	10	9.7
ADHD, any	24	23.3
Borderline Personality Disorder	29	28.2
Other diagnosis^b^	27	26.2

^a^ Not mutually exclusive categories

^b^ Conduct disorder, Oppositional defiant disorder, Unspecified disruptive behaviour disorder, Chronic motor or Vocal tic disorder, Autism spectrum disorder, Adjustment disorder. ADHD = Attention-deficit/hyperactivity disorder

^c^One or both parents born in Afrika (3.9%); Asia (15.5%) South/Central-America (9.7%); Eastern/Southern-Europe (6.8%); Northern-Europe (5.8%/)

### Characteristics of the self-harm behaviour

Distribution of self-harm characteristics and selected covariates are presented in Table 2. The median age of self-harm onset was 13.2 years (range 7.1–17.4), and the median duration of self-harm was 2.5 years (range 0.1–10.3) (Fig. 1a). The lifetime frequency of NSSI episodes ranged from 0 to 990 (median = 50; IQR = 126; 75 percentile = 142). Only one adolescent reported having had no NSSI episodes, but this participant reported repeated suicide attempts. The lifetime number of methods used for self-harm varied from 1 to 10. Cutting with a sharp object was the most commonly reported NSSI method (93.2%), followed by self-battery (41.7%), and stabbing (16.3%). Fifty adolescents (48.5%) reported at least one lifetime suicide attempt, of which twenty-seven (26.2% of the total sample) repeated the attempt at least once, with cutting (21.4%), drug overdose (21.4%) and asphyxia (14.6%) as the most frequently reported methods.

**Table 2:** Self-harm characteristics and selected covariates in adolescents with repetitive self-harm (*N* = 103)

	Mean	Std.Dev	Median	Interquartile range
**Self-harm characteristics**				
Age of onset (years)	13.1	1.8	13.2	1.9
Duration (years)	2.8	2.1	2.5	2.9
Suicide attempts, lifetime	1.8	4.4	0.0	2.0
NSSI, lifetime	119.7	174.3	50.0	126.0
Duration before first SA	2.2	1.9	1.6	6.5
NSSI before first SA	89.7	134.9	34.5	90.0
Number of methods (first SH episode)	1.1	0.2	1.0	0.0
Number of methods (lifetime)	2.7	1.6	2.0	2.0
**Selected covariates** ^a^				
Borderline Symptoms (BSL-23)	35.15	23.07	31.0	39.0
Sexual abuse	0.31	0.46	0	1.0
Physical abuse	0.33	0.47	0	1.0
Relationship with mother	32.83	10.15	35.0	17.0
Relationship with father	31.50	10.20	33.0	12.5

*SA* Suicide attempt, *SH* Self harm

^a^ Potential confounders

### Age of self-harm onset and duration of self-harm by frequency of NSSI

A 3D-barplot of the median lifetime NSSI episodes in three categories of age of onset against four categories of duration of self-harm behaviour is shown in Fig. 1c. For each category of age of onset, an increasing median lifetime frequency of NSSI episodes was observed for increasing duration of self-harm, except for high values of age of onset and duration. The adjusted regression analysis, (Table 3), showed that age of onset, interaction between age of onset and duration, and their squared terms all remained significantly associated with the frequency of NSSI episodes.

**Table 3:** Negative binomial regression model for NSSI lifetime episodes with age of self-harm onset, duration of self-harm and selected covariates, in adolescents with repetitive self-harm (*N* = 103)

	Estimate	St. Error	z value	*p*-value	95% CI	
(Intercept)	2.95	0.43	6.82	***	2.07	3.91
Relationship with mother	−0.03	0.01	−2.30	*	−0.05	−0.01
Physical abuse	−0.12	0.25	−0.47	n.s	−0.68	0.46
Sexual abuse	0.43	0.26	1.68	n.s	− 0.10	0.98
Age of onset	−0.19	0.09	−2.07	*	−0.39	−0.01
Duration of self-harm	0.16	0.09	1.82	0.068	−0.01	0.33
Age of onset - squared	−0.19	0.06	−3.35	***	−0.31	−0.10
Duration - squared	−0.19	0.06	−3.48	***	−0.31	−0.10
Age of onset x Duration (Interaction)	−0.36	0.10	−3.55	***	−0.58	−0.13

*p < .05; **p < .01; ***p < .001

**Fig. 2:**
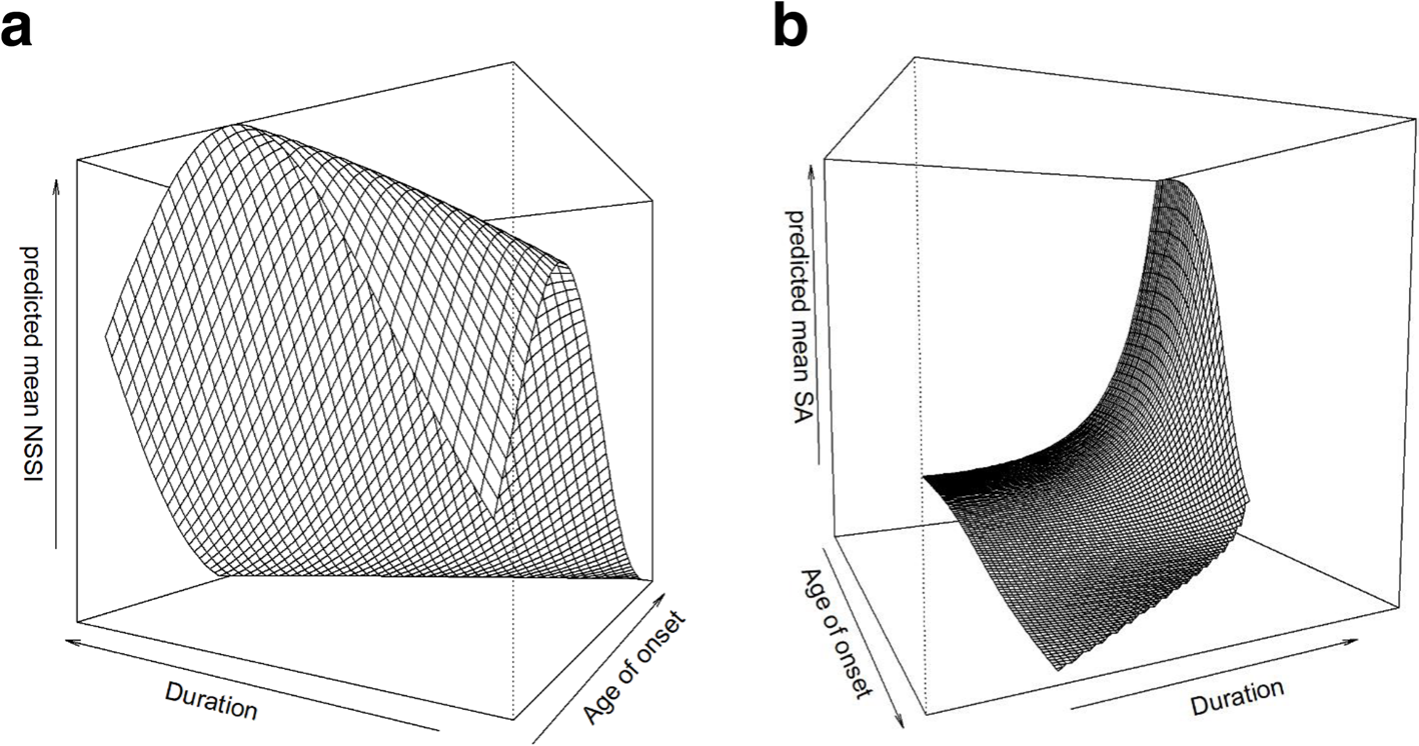
**a** Predicted mean of NSSI lifetime episodes (z-axis, range: 0–524) from negative binomial regression model on grid of age of self-harm onset and duration of self-harm in adolescents with repetitive self-harm (*N* = 103). **b** Predicted mean of suicide attempts lifetime (z-axis, range: 0–10) from hurdle regression model, on grid of age of self-harm onset and duration of self-harm in adolescents with repetitive self-harm (N = 103)

The predicted mean frequency of mean NSSI episodes were plotted against age of self-harm onset and duration of self-harm (Fig. 2a). The figure shows that there was an increase in frequency of NSSI episodes for decreasing age of onset and increasing duration, except for short duration, where the frequency of NSSI increased and then decreased for lower values of age of onset. The drop in the front of the plot for small values of age of onset and duration of self-harm in Fig. 2a corresponds with the upper and inner left corner in Fig. 1c.

### Age of self-harm onset and duration of NSSI on risk by suicide attempts

A Kaplan-Meyer plot of age at first suicide attempt, stratified by three categories of age of onset of self-harm (Fig. 1b), illustrates that an early age of self-harm onset was associated with an earlier first suicide attempt (unadjusted). Adjusted regression results (Table 4) show that both age of onset and duration of NSSI before first suicide attempt, separately and in interaction with each other, were significantly associated with probability of a first suicide attempt. With regard to repeated suicide attempts, duration of NSSI before the first suicide attempt was strongly associated with increased frequency of suicide attempts and borderline significant (*p* = 0.059). Age of onset was not significantly associated with repeated suicide attempts in this analysis.

**Table 4:** Fitted two-component hurdle model for first suicide attempt (logit link) and repeated suicide attempts (SA) (log link) with age of self-harm onset, duration of self-harm and selected covariates, in adolescents with repetitive self-harm (N = 103)

	**First suicide attempt**					
	Estimate	St. Error	z value	p-value	95% CI	
(Intercept)	−2.04	0.54	−3.78	***	−3.10	− 0.98
Borderline Symptoms (BSL-23)	0.05	0.01	3.77	***	0.02	0.07
Age of onset	−0.67	0.21	−3.20	**	−1.09	−0.26
Duration before first SA	−0.63	0.20	−3.25	**	−1.02	−0.25
Age of onset x Duration before first SA (Interaction)	−0.19	0.08	−2.37	*	−0.34	−0.03
	**Repeated suicide attempts**					
	Estimate	Std. Error	z value	p-value	95% CI	
(Intercept)	−5.64	0.95	−5.97	***	−7.50	−3.80
Borderline Symptoms (BSL-23)	0.04	0.01	4.03	***	0.02	0.07
Sexual abuse	1.13	0.52	2.19	*	0.12	2.15
Duration before first SA	−0.09	0.12	−0.74	n.s	−0.33	0.15
Duration before first SA - squared (interaction)	0.15	0.078	1.89	0.059	−0.01	0.30

*p < .05; **p < .01; ***p < .001

Both age of onset and duration of self-harm were significantly associated with the expected frequency of suicide attempts, in the total hurdle model (Fig. 2b). The figure shows that the predicted mean frequency of suicide attempts increased with increased duration of NSSI before first suicide attempt, for all low and moderate values of age of onset, but dramatically more for lower age of onset. In addition, the frequency of suicide attempts increased with decreasing age of onset when the duration of NSSI before first suicide attempt increased, and dramatically more for longer duration before first suicide attempt.

The selected covariates were considered potential confounding factors, and were adjusted for in all analysis. With respect to frequency of NSSI episodes, a positive relationship with mother was significantly associated with a decrease in the frequency of NSSI and with increasing age of onset (Table 3). Higher scores on borderline symptom list (BSL-23) were significantly associated with earlier age of onset, longer duration of self-harm and higher risk of a first suicide attempt, as well as repeated suicide attempts (Table 4), while, sexual abuse was significantly associated with lower age of onset and increased frequency of suicide attempts.

## Discussion

Our study is the first to examine whether age of onset, uniquely and in interaction with duration of self-harm is associated with frequency of NSSI and risk of suicide attempts in a clinical adolescent population. The main findings support our hypothesis and were that both earlier age of onset and longer duration were associated with increased frequency of NSSI and suicide attempts.

### Age of onset, duration and NSSI

Our findings are compatible with findings in adult populations [9, 10] indicating that an earlier age of onset is a potential risk factor for increased lifetime frequency of NSSI, even when controlled for duration and selected covariates. It would seem self-evident that a longer duration of self-harm leads to more NSSI episodes irrespective of the age of onset. However, we found that among adolescents with similar duration but different age of onset, the highest frequency of NSSI episodes was found in those who started at an earlier age.

We also found that a positive relationship with mother was significantly associated with a decrease in the frequency of NSSI episodes and increasing age of onset. This finding corresponds with previous studies indicating that perceived family support predicted termination of NSSI, and that negative perceptions of parents were related to a higher frequency of NSSI [16, 38].

### Age of onset, duration and suicide attempts

About half of our participating adolescents reported having had at least one suicide attempt. Suicide attempts were more likely in participants with a lower age of self-harm onset and a longer duration. This is in line with recent research on adults [10, 39] indicating that the risk of suicide attempts increases with earlier age of self-harm onset. These studies are, however, based on adult samples and are not readily comparable with a clinical sample of adolescents.

Mental disorders are associated with an increased risk of suicidal behaviour in adolescents [19]. In addition, mental illness combined with ongoing self-harm, further increases risk of suicide attempts [3]. In our study, the average number of DSM-5 diagnoses was 3.2, (range 0–8), and 84.5% reported symptoms consistent with a depressive disorder. However, we did not find significant associations between early age of onset and number of diagnoses, nor between age of first depressive episode and suicide attempts (preliminary analyses).

### Age of onset, duration and repeated suicide attempts

About a fourth of participating adolescents reported repeating the suicide attempt at least once. When we examined the expected number of suicide attempts, we found an increase in suicide attempts when the age of onset decreased and the duration increased, and dramatically more for long duration of NSSI before first suicide attempt. This is consistent with a possible direct effect of age of onset. This would mean that if you compare two adolescents with self-harm behaviour and equal duration of NSSI (before the first suicide attempt), it would be the adolescent with earlier age of onset that has the highest expected number of suicide attempts. Furthermore, the number of suicide attempts increased with longer duration of NSSI (before the first suicide attempt) for early and moderate onset age, and dramatically more for early onset age (possible effect modification from age of onset on the effect of duration). Likewise, if you compare two adolescents, with self-harm behaviour and equal age of self-harm onset, then based on our findings, the adolescent with the longer duration of NSSI (before the first suicide attempt) would have the highest expected number of suicide attempts.

These results are in line with previous research suggesting that longer duration of NSSI is associated with increased risk of suicide attempts [40, 41], that suicide attempts are one of the most important risk factors for re-attempts [42], and that the highest risk of re-attempts is relatively shortly after the first suicide attempt [42]. A possibility as to why NSSI elevates the risk of suicide attempts was made by Joiner (2005). He hypothesised that the transition from NSSI to suicide attempts may be explained by an acquired capability for suicide attempts through repetition of self-harm over time [43]. Studies that confirm this theory find that increased risk of suicide attempts is associated with reduced pain and fear [40], and an increased belief in own capabilities to do what is needed [44]. Our findings are of clinical relevance and suggest that strategies of early assessment and treatment in this group of adolescents is particularly important to reduce current and future suicidal behaviour [45].

In addition, we found that a higher level of borderline symptoms among these adolescents was associated with an increased risk of suicide attempts (first and re-attempts). This finding corresponds with previous studies indicating that borderline symptoms is associated with greater severity of self-harm [46] and suggests that it would be important to assess borderline symptoms in adolescents with recurrent self-harm behaviour as a part of suicide risk assessment and management [47–49]. Certainly, it could assist clinicians in selecting treatments specifically designed to address these symptoms and challenges in adolescents [45, 50–52]. Finally, we found that having been sexually abused was significantly associated with an increased risk of repeated suicide attempts. Due to small numbers, this finding must be interpreted with caution, but it corresponds well with numerous other studies having made the same finding [3].

### Strengths and limitations

A strength of this study is that it is based on a clinical sample of adolescents with recurrent self-harm. Among limitations are the study’s cross-sectional design precluding causal inference, and the retrospective retrieval of information entailing risks of memory and information-biases. The frequency of self-harm episodes, especially for those with long duration, is an estimate. According to the limited number of males included in the sample preventing us from generalizing these findings to males. Due to the nature of the age inclusion criteria, we lack data beyond older age of onset for the oldest adolescents. This is natural given the time horizon of the current study, and is important to take into consideration while interpreting these results. Future studies with more diverse samples and with a longitudinal design would improve confidence in the results, as well as generalizability.

## Conclusions

Despite the mentioned limitations, our study suggests that both an early age of self-harm onset and a longer duration of self-harm may entail an increased frequency of further NSSI and suicide attempts in adolescents with recurrent self-harm, and that the youngest are particularly at risk of increased self-harm, especially when the duration increases. Recurrent self-harm over time may lead to an acquired capability for suicide attempts, and emphasizes the importance of early detection and interventions adapted to both age and severity.

## Data Availability

The datasets analysed during the current study are available from the corresponding author on reasonable request.

## Abbreviations

NSSI: Non-Suicidal Self-Injury
SA: Suicide Attempt
SH: Self-Harm
BSL-23: Borderline Symptom List
